# A rare case of S*treptococcus sanguinis* mycotic popliteal aneurysm

**DOI:** 10.1099/jmmcr.0.001479

**Published:** 2014-12-01

**Authors:** Karan Jolly, Rachel Barratt, Amit Nair

**Affiliations:** ^1^​ Russells Hall Hospital, Dudley DY1 2HQ, UK

**Keywords:** Mycotic aneurysm, popliteal aneurysm, *Streptococcus sanguinis*, vascular surgery

## Abstract

**Introduction:**

Mycotic popliteal aneurysms are not a common phenomenon. They can initially be easily confused with other more trivial conditions such as a Baker’s cyst. We present a case of a patient presenting with a progressively worsening leg swelling which was initially misdiagnosed. Only until symptoms rapidly progressed was a popliteal aneurysm diagnosed. To our knowledge this is the only identified case of a *Streptococcus sanguinis* mycotic popliteal aneurysm.

**Case presentation:**

An 81-year-old gentleman presented to the surgical assessment unit with a six-week history of a painful, diffuse swelling in the left popliteal fossa. Initially, when symptoms developed a provisional diagnosis of a Baker’s cyst was made. When the symptoms progressed to involve swelling of the entire lower limb, an ultrasound was arranged. Detailed Imaging revealed a popliteal aneurysm with signs of rupture. Urgent repair was performed, with high suspicion of a mycotic aneurysm intra-operatively. Cultures confirmed this, isolating *Streptococcus sanguinis*. Multiple investigations failed to isolate an acute infective source of this infection. The patient recovered promptly with a long course of intravenous antibiotics, being able to mobilize normally.

**Conclusion:**

Mycotic popliteal aneurysms are not very common and can easily be confused with other benign lesions. The key to diagnosis is the presence of a pulsatile mass and further detailed imaging. This case was unique in that *Streptococcus sanguinis* has not been isolated from such an aneurysm until now. The most likely explanation of this case was that the aneurysm was secondary to transient bacteraemia of this organism through the oral cavity, in the absence of any cardiac involvement.

## Introduction

Mycotic popliteal aneurysms are not a common phenomenon. They can initially be easily confused with other more trivial conditions such as a Baker’s cyst. We present a case of a patient presenting with a progressively worsening leg swelling which was initially misdiagnosed. Only when symptoms rapidly progressed was a popliteal aneurysm diagnosed. To our knowledge, this is the only identified case of a *Streptococcus sanguinis* mycotic popliteal aneurysm.

## Case report

An 81-year-old gentleman presented acutely to the surgical assessment unit with a painful, diffuse swelling in the left popliteal fossa. The swelling, which was first noticed 6 weeks prior to admission, came on suddenly and was associated with pain and tenderness. Over the following weeks, it had progressed in size and become increasingly painful. The patient had a background history of hypertension, but was not on any regular medication.

A provisional diagnosis of a Baker’s cyst had been made by the patient’s primary health care provider and he had subsequently been awaiting an Orthopaedic clinic appointment for this. The swelling continued to increase in size, restricting the range of movement at the knee. The patient subsequently attended the emergency department where a pulsatile popliteal fossa mass was found. The impression was still of a Baker’s cyst and the patient was sent home. In the weeks to follow, the patient struggled to bear weight on the left leg. The 24 hours leading up to his present admission was accompanied by a sudden onset diffuse swelling in the entire left lower leg. An urgent ultrasound was arranged to exclude a deep venous thrombosis which instead revealed a large, 8 cm, popliteal aneurysm in the popliteal fossa, the outer wall of which was not well defined. There was a 5 cm patent channel noted within the centre of this structure with extensive intramural thrombus noted at the periphery. No obvious abdominal aortic aneurysm was seen.

**Fig. 1.**
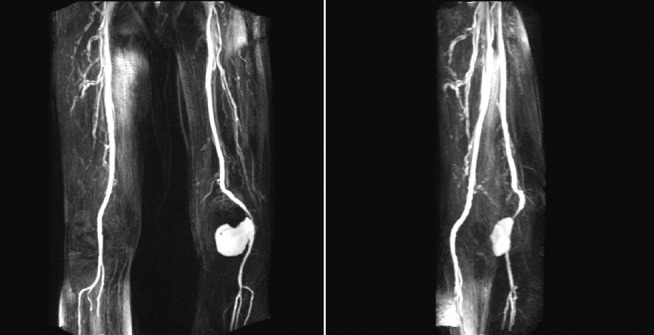
MRA revealing a left-sided popliteal aneurysm.

On initial surgical evaluation, the patient was haemodynamically stable and apyrexial. The left leg was grossly oedematous from below the knee, with a pulsatile firm tender mass in the popliteal fossa. The pedal pulses were all palpable with good range of movement in the feet; however, there was marked reduction of movement at the knee joint. Initial blood tests including white cell count and C-reactive protein were normal. To get a more detailed image of the aneurysm and arterial tree in the left leg, an urgent magnetic resonance (MR) arteriogram was arranged, which showed a false aneurysm of the popliteal artery measuring 8.8 cm coronally and 7.4 cm axially (Fig. 1). Approximately 50% of the false aneurysm was thrombosed and distal runoff was well preserved. No other aneurysm or stenosis was found elsewhere in the vasculature of the lower limbs. A decision was made to immediately perform an emergency open repair due to rapid clinical deterioration, possibly due to an acute bleed.

Per-operatively, the aneurysm was approached posteriorly and the sac dissected. Bleeding was controlled and clamps applied at either end of the aneurysm. The appearance of the aneurysm was very much indicative of a mycotic aneurysm, and a tissue sample was sent to the microbiology laboratory for microscopy and culture. A 5 cm vein graft was used to repair the aneurysm and the wound was closed. After the procedure, a strong flow signal was detected distal to the aneurysm repair. In the days to follow the patient recovered very well and the oedema in the leg slowly started to settle.

The aneurysm tissue was processed using the local Microbiology Laboratory Standard Operating Procedure for sterile tissues. It was emulsified in sterile saline using sterile glass beads, then plated onto 5% sheep blood (BA), chocolate (CA), CLED and anaerobic (ABA) agar plates and into cooked meat broth (CMB) (bioMérieux), which were incubated in appropriate atmospheres at 37 °C for 48 h (5 days for the anaerobic plate and CMB). The culture on CMB was sub-cultured after 5 days onto BA, CA and ABA, which were then incubated as above. The isolate was identified as *Streptococcus sanguinis* by API 20 Strep (bioMérieux) – a member of the viridans group streptococci. These results were available on the seventh day post-operatively, confirming the suspicion of a mycotic aneurysm. Intravenous benzylpenicillin and flucloxacillin were commenced for 2 weeks, as recommended by the microbiologist.

Despite these findings, the patient remained afebrile and serial blood cultures taken all came back negative. As the organism is commonly part of the oral flora, an orthopantomogram and transthoracic echocardiogram (TTE) were requested to exclude infective endocarditis. Both of these tests were inconclusive and the patient subsequently refused to have a *trans*-oesophageal echocardiogram (TOE) to evaluate his heart further. The patient regularly visited his dentist and had reasonable dental hygiene. In the absence of any focal source of sepsis, a conclusion was made that the mycotic aneurysm was secondary to transient *Streptococcus sanguinis* bacteraemia through the oral mucosa. The patient continued to show significant improvement in mobility in the weeks to follow.

## Discussion

Mycotic aneurysms were historically defined as infected aneurysms developing within a previously normal artery secondary to septic embolization due to bacterial endocarditis (Wilson *et al.* 1978). The absence of fungal organisms in the pathogenesis of this process, however, has led to many adopting the term proposed by Jarrett *et al.* (1975), ‘infected aneurysm’. In accordance with Osler’s first description in 1885 of a mushroom-shaped aneurysm in a patient with bacterial endocarditis, an aneurysm with a saccular morphology is indicative of an infective process (Osler 1885).

Mycotic popliteal artery aneurysms (MPAA) are a rarely described phenomenon and have only been reported approximately 50 times in the English literature. Despite the popliteal artery being the most common peripheral site for aneurysm formation, mycotic aneurysms tend to favour the femoral artery and abdominal aorta (Joffe *et al.*, 1996).

It has a male preponderance, estimated at 11:3 (male:female), with no particular age predilection and a mean age of onset at 41 years (Wilson *et al.*, 1978). MPAA are typically caused by septic emboli (historically from bacterial endocarditis) in the vasa vorum or lumen of peripheral arteries. Other reported pathological processes included extension of infection to the vessel from a contiguous infective process, for example septic arthritis, and direct inoculation of the vessel with an infective organism, for example in trauma or direct arterial injury. The resulting ischaemia of vessel wall or vasa vorum from infection leads to destruction of the normal structure of the vessel and aneurysm formation (Stengel & Wolferth, 1923). Whilst Wilson’s definition implies that MPAA occurs ‘within a previously normal artery’ this applies only to the previous lack of an aneurysm in the vessel. Indeed it is unusual for mycotic aneurysms to occur in healthy arteries unless the patient is additionally immunocompromised or the organism particularly virulent.

Reviews of the available literature suggest that, in many cases, blood cultures do not identify a causative organism. However, when the organism has been identified, *Staphylococcus aureus* is the most prevalent cause of MPAA followed by *Streptococcus viridans* and *Staphylococcus epidermidis*. Other organisms are varied and include *Campylobacter jejuni*, *Escherichia coli*, *Streptococcus faecalis* and *Streptococcus pneumoniae*, and *Tuberculosis* and *Salmonella* spp. (Killen *et al.*, 2009). This is the first reported case of MPAA caused by *Streptococcus sanguinis* to the best of our knowledge. *Streptococcus sanguinis* is a Gram-positive cocci, a normal colonizer of the oral cavity, and is thought to be protective against dental caries (Caufield *et al.*, 2000). It is also a common causative agent in bacterial endocarditis, especially in patients who have undergone recent dental work. It is perhaps surprising, given its prevalence in bacterial endocarditis, that this organism has not been identified before in a MPAA. However this is likely due to the relative rarity of MPAA.

Unfortunately for clinicians, mycotic popliteal artery aneurysms have a varied and inconsistent presentation. That said, there are some features that one would expect to find in a typical MPAA. Classically, MPAA present as a painful, tender, pulsatile swelling in the popliteal fossa. Some patients will also be pyrexic and often have a primary infective focus such as bacterial endocarditis. As with our case, MPAA can be confused with a Baker’s Cyst and further evaluation is not sought until symptoms of the aneurysm itself, such as claudication, acute ischaemia or digital thromboembolism arise (Varga *et al.*, 1994).

Laboratory studies typically show raised inflammatory markers and a leukocytosis but this is not present in all patients. Blood cultures, as previously mentioned, are only present in approximately 50% of reported MPAA cases, although this may be explained by pre-diagnosis treatment with antibiotics for the primary infective focus (Killen *et al.*, 2009). Imaging is diagnostic for MPAA and modalities include colour-duplex ultrasound, computed tomography (CT) angiogram, MR angiogram or, more historically, conventional interventional angiogram. CT angiogram and MR angiogram are the favoured imaging modalities used in current practice. Imaging provides the diagnostician with information regarding size, diameter and morphology; as mentioned, saccular morphology is highly suggestive of an infective pathophysiology.

Once identified, two issues must be addressed: firstly identifying and treating the primary infective source and secondly treating the popliteal aneurysm itself. For the former, this involves assessment of the entire cardiovascular system and often a transoesophageal echocardiogram regardless of the presence of an audible heart murmur. If this is negative it is also worth investigating the adjacent knee joint for the presence of a septic effusion and taking a history for recent trauma or damage to the knee joint. Both of these conditions are treated in the main by at least 2 weeks of intravenous antibiotics followed by 4 weeks of intravenous or oral antibiotics according to clinical response. Septic arthritis also involves arthroscopy or open washout of the knee joint. Treatment of the popliteal aneurysm itself and any complications that have ensued is normally guided by multidisciplinary team management but in the main consists of resection and revascularization. At present, there is no evidence-based consensus on the approach for popliteal aneurysms with both medial and posterior approaches being utilized (Safar & Cina, 2001). The aneurysm itself is resected or excluded from circulation and the artery revascularized with standard reconstructive techniques using an autologous vein graft (normally long saphenous vein) (Killen *et al.*, 2009; Safar & Cina, 2001). Synthetic grafts are avoided due to the presence of infection (Killen *et al.*, 2009; Varga *et al.*, 1994). At least two case reports describe the use of endovascular techniques to manage a ruptured infected popliteal artery aneurysm, both with satisfactory short- to medium-term outcomes. Whilst there is the benefit of endovascular treatment being minimally invasive, this has to be balanced with the risk of implanting synthetic graft material in an infected space (Schimmer & Somjen, 2009;Bani–hani *et al.*, 2012). This technique may become more popular with recently published improved patency rates for non-mycotic popliteal artery aneurysms treated endovascularly (Bani–hani *et al.*, 2012).

Occasionally MPAA, as with all popliteal aneurysms, can result in the need for amputation either as a result of escalation of the primary process itself prior to treatment or failure of the graft. It is likely that these cases are underreported.

## Summary

MPAA and indeed all mycotic aneurysms are much rarer nowadays due to the high index of suspicion for bacterial endocarditis in patients with heart murmurs followed by early and aggressive antibiotic treatment. However, despite their rarity this case report has illustrated new bacteriology in its pathogenesis and also highlighted the pitfalls in diagnosis of the condition.

## Abbreviations

MPAA: mycotic popliteal artery aneurysm
MR: magnetic resonance
